# Associations Between Temperament and Dental Caries Status in Thai Children

**DOI:** 10.3290/j.ohpd.a43350

**Published:** 2020-04-03

**Authors:** Oitip Chankanka, Songchai Thitasomakul, Angkana Thearnmontree, Supatcharin Piwat, Wattana Pithpornchaiyakul, Benjaporn Panyayong

**Affiliations:** a Assistant Professor, Preventive Dentistry Department, Children Oral Health Promotion and Caries Prevention Research Unit, Faculty of Dentistry, Prince of Songkla University, Hat Yai, Songkhla, Thailand. Hypothesis, study design, data collection, statistical analysis and manuscript writing.; b Associate Professor, Preventive Dentistry Department, Children Oral Health Promotion and Caries Prevention Research Unit, Faculty of Dentistry, Prince of Songkla University, Hat Yai, Songkhla, Thailand. Idea, study design, data collection and discussion.; c Assistant Professor, Preventive Dentistry Department, Faculty of Dentistry, Prince of Songkla University, Hat Yai, Songkhla, Thailand. Study design, data collection and discussion.; d Assistant Professor, Preventive Dentistry Department, Faculty of Dentistry, Prince of Songkla University, Hat Yai, Songkhla, Thailand. Study design and data collection.; e Assistant Professor, Preventive Dentistry Department, Faculty of Dentistry, Prince of Songkla University, Hat Yai, Songkhla, Thailand. Responsible for data collection.; f Psychiatrist, Department of Mental Health, Ministry of Public Health, Nonthaburi, 11000 Thailand. Study design and data collection.

**Keywords:** dental caries, temperament, primary dentition, child

## Abstract

**Purpose:**

To examine the relationships between (1) the levels of each temperament traits and the levels of milk-feeding frequency, oral hygiene care and dental caries, and (2) the difference in mean numbers of decayed surfaces among temperament types.

**Materials and Methods:**

Four hundred and ninety-three (493) 12-month-old infants were assessed for temperaments and characteristics of child-rearing practices. The data were analysed with their dental caries status at 18 months of age. The chi-squared test, Student’s t test, ANOVA and Kruskal-Wallis tests were used to assess the association between temperament traits and the following variables; dental caries, oral cleaning habits and feeding frequency. Logistic regression models were used to identify the effect of temperament traits and other factors on dental caries status.

**Results:**

The trait of adaptability was found to associate with feeding frequency at night, while those of activity and approach/withdrawal were significantly associated with regularity of oral cleaning habits by the children’s caregiver. Three of the nine temperament traits – biological rhythmicity, approach/withdrawal and mood – were significantly associated with dental caries in bivariate analysis. Biological rhythmicity and approach/withdrawal traits were significantly associated with a higher chance of having caries after adjusting for regularity of oral cleaning habits and frequency of night feeding.

**Conclusion:**

Biological rhythmicity, approach/withdrawal and mood traits were related to caries in these young children.

Early childhood caries (ECC) is a common disease in children, especially in developing countries. The 7th National Oral Health Survey in 2011 in Thailand showed that 51.7% of 3-year-old children had caries.^1^ Dental caries in infants and toddlers have been found to be related to parental child-rearing practices, including child feeding habits and children’s oral hygiene care.^6,8,11–13,20^ Parents who usually do not brush their children’s teeth and/or use milk feeding to soothe their children to sleep, complain about their children’s difficult/uncooperative behaviours. Therefore, the question was raised whether temperament affects caries risk in children.

Temperament presents at birth and continues to influence children’s development in many ways throughout their lives. The New York Longitudinal Study by Thomas and Chess identified nine temperament characteristics and classified three types of temperament as ‘difficult’, ‘easy’, or ‘slow-to warm-up’ with some unclassified children.^19^ To identify children’s temperament, individual differences in children’s reaction to varied situations and circumstances are measured.

Only a few studies have assessed the associations between child temperament and dental caries. The three studies were conducted in North America. One study compared temperament between children with and without caries.^9^ No difference was found in the nine temperament component scores and temperament clusters between the two groups of children. Quinonez and colleagues assessed the associations between ‘strong tempered’ children, parent feeding practices and ECC.^15^ They found that a combination of being a native Canadian, a high level of shyness and greater duration in feeding habits predicted ECC. However, there was no association between temperament and the duration of feeding. A study among children in Iowa City found a relationship between mothers’ perceptions about their children’s temperament and prevalence of non-cavitated caries in their bivariate analysis.^17^

A recent study, conducted in Iran, compared different traits of temperament in children with and without caries, which assessed the associations between temperament and nutritional and oral hygiene habits.^2^ They reported that attention shifting, frustration and shyness were risk factors for caries. Based on previous published studies, there are inconsistences in the findings among groups of different races and cultures. Different countries have different ways of child rearing, which are associated with their cultures, for example, co-sleeping, being in an extended family and methods used to induce children to sleep. It is interesting to find out whether temperament has any effect on oral health related issues and dental caries in children. Thus, the purposes of this study were to examine: (1) the associations between the levels of each individual temperament trait, and the levels of milk-feeding frequency, oral hygiene care and dental caries, and (2) the difference in mean numbers of decayed surfaces among temperament types.

## MATERIALS AND METHODS

This longitudinal community-based study is associated with the national child health research, Prospective Cohort Study of Thai Children (PCTC), aiming at following up infants from birth to 24 years of age. Thepa, the selected district where this study was conducted, is in the southern region of Thailand. A cohort of those born between the 1-year period from 2000 to 2001 was included.

The present study was approved by The Ethics Committee of the Faculty of Dentistry, Prince of Songkla University. A cluster sampling technique was used to select 795 members of the birth cohort from 8 out of the 12 health centres.

The PCTC team used the 36 items of a self-reporting questionnaire translated from the Australian Toddler Temperament Questionnaire with a back translation check to interview mothers to obtain temperament data when the children were 12 months of age.^7^ Four or five items of the questionnaire were used for each of the nine temperament traits. The answers were decoded to numbers (never = 1 to usually = 4), which were then calculated within each trait. The scores of each temperament trait were used to categorise the children into high (fourth quartile), normal (second and third quartiles) and low (first quartile) score levels. The meaning of the temperament characteristics is described in Table 1.^14^ The scores of the temperament characteristics were also used to classify into four temperament types (easy, difficult, slow to warm up and unclassified types). At the same time, the dental team used the questionnaire to interview the mothers regarding milk-feeding methods (breast feeding, bottle feeding or both), frequency of day- and night-time milk feeding, and method and frequency of teeth cleaning.

**Table 1:** Descriptions for low and high scores of each temperament trait

Dimension/Trait	Description	Low score	High score
Activity	Motor activity	Not active	Very active
Rhythmicity	Regularity of behaviours such as sleeping and feeding	Rhythmic, regular	Arrhythmic, irregular
Approach-withdrawal	Response to a new person/stimulus	First response is approach	First response is withdrawal
Adaptability	Adaptation to changes environment	Very adaptable	Not adaptable
Intensity	Level of energy in responding or reacting	Not intense	Very intense
Threshold of responsiveness	Stimulation strength necessary to arouse a discernible response	High threshold	Low threshold
Mood	Contrast between amount of friendly/ happy behaviour and unfriendly/unhappy behaviour	Positive, happy	Negative, unhappy
Distractibility	Level of extraneous stimuli which alters ongoing behaviour	Not distractible	Distractible
Persistence or attention span	Amount of time spent on an activity, and the distraction effect on the activity	Persistent, long attention span	Not persistent, short attention span

From Prior et al, 1987.^14^

At 18 months of age, the children were orally examined by five trained and calibrated dentists using modified WHO criteria (1997). The caries lesions were recorded at the surface level, which included four surfaces of the anterior and five surfaces of posterior teeth. More details of the dental examinations were published elsewhere.^18^ The scoring system was modified from the WHO criteria, 1997. The dental status for each surface was coded using the following: U for unerupted surface; S for normal tooth surface; d1 for initial caries/caries limited to enamel both with/without cavity; d2 for dentine caries; and d3 for caries involving pulp (a deep lesion probably pulpal involvement or with present/history of pain/swelling/fistula opening). Before data collection, five examiners and recorders learnt and discussed the process of data collection including the dental examination criteria. Then the calibration was carried out at the university day care centre. The Cohen’s Kappa for intraexaminer reliability ranged from 0.75 to 0.91, and for interexaminer reliability the values were from 0.68 to 0.89.

Chi-squared tests were used to assess the association between temperament traits, dental caries status (caries, caries free) and oral cleaning habits (regularly, sometimes/never). The Student’s t test, ANOVA and Kruskal-Wallis tests were used to compare temperament traits with ds and feeding frequency. Multivariable logistic regression models were used to identify temperament traits and other factors associated with dental caries status (caries, caries-free). All analyses were conducted using SPSS 17.0 (SPSS, Chicago, IL, USA). Statistical significance was set at p < 0.05.

## RESULTS

A total of 493 children were dentally examined and interviewed; 50.3% were boys. At 18 months of age, 29% of these children had d2 and/or d3 caries and 66.7% of the children had dental caries at any level (d1, d2 and/or d3).

Table 2 shows the percentage of children with caries and the mean number of carious surfaces by the children’s level of each temperament trait. Levels of rhythmicity, approach/withdrawal and mood traits were significantly associated with the proportion of children with caries (p < 0.05). The results also show that children with a high-score level of rhythmicity (arrhythmic) had significantly greater numbers of decayed surfaces compared to those with normal-score and low-score levels (p < 0.05). In addition, children with a low-score level of mood (positive/happy) had a significantly lower number of decayed surfaces compared to those with normal-score and high-score levels (p < 0.01).

**Table 2:** Association of dental caries status and/differences of mean caries surfaces by temperament trait score levels

Temperament trait	Level	n	n (%)		Mean caries surfaces (± SD)
			Caries	Caries-free	
Activity	Low	154	100 (64.9%)	54 (35.1%)	5.25 (± 5.91)
	Normal	196	127 (64.8%)	69 (35.2%)	4.95 (± 6.07)
	High	143	102 (71.3%)	41 (28.7%)	4.74 (± 5.42)
Rhythmicity*, **	Low	139	88 (63.3%)	51 (36.7%)	4.71 (± 5.77)
	Normal	222	141 (63.5%)	81 (36.5%)	4.43 (± 5.40)
	High	132	100 (75.8%)	32 (24.2%)	6.19 (± 6.43)
Approach/withdrawal*	Low	124	78 (62.9%)	46 (37.1%)	4.79 (± 5.61)
	Normal	157	95 (60.5%)	62 (39.5%)	4.55 (± 5.61)
	High	212	156 (73.6%)	56 (26.4%)	5.41 (± 6.11)
Adaptability	Low	139	86 (61.9%)	53 (38.1%)	4.16 (± 5.12)
	Normal	157	109 (69.4%)	48 (30.6%)	5.52 (± 6.41)
	High	197	134 (68.0%)	63 (32.0%)	5.14 (± 5.78)
Intensity	Low	207	138 (66.7%)	69 (33.3%)	5.05 (± 6.06)
	Normal	83	60 (72.3%)	23 (27.7%)	4.61 (± 5.01)
	High	203	131 (64.5%)	72 (35.5%)	5.07 (± 5.92)
Threshold of responsiveness	Low	128	81 (63.3%)	47 (36.7%)	4.69 (± 5.55)
	Normal	187	123 (65.8%)	64 (34.2%)	4.81 (± 6.01)
	High	178	125 (70.2%)	53 (29.8%)	5.38 (± 5.85)
Mood*, **	Low	179	109 (60.9%)	70 (39.1%)	3.92 (± 4.95)
	Normal	178	133 (74.7%)	45 (25.3%)	5.62 (± 6.04)
	High	136	87 (64.0%)	49 (36.0%)	5.54 (± 6.44)
Distractibility	Low	194	124 (63.9%)	70 (36.1%)	5.57 (± 6.35)
	Normal	134	95 (70.9%)	39 (29.1%)	5.33 (± 6.08)
	High	165	110 (66.7%)	55 (33.3%)	4.02 (± 4.81)
Persistence or attention span	Low	154	100 (64.9%)	54 (35.1%)	4.95 (± 5.97)
	Normal	160	102 (63.8%)	58 (36.2%)	4.81 (± 5.77)
	High	179	127 (70.9%)	52 (29.1%)	5.17 (± 5.79)

Note: *for p <0.05 by chi-square test and ** for p <0.05 by ANOVA/Kruskal-Wallis test; mean decayed surfaces including d1, d2 and d3 caries.

**Fig 1:**
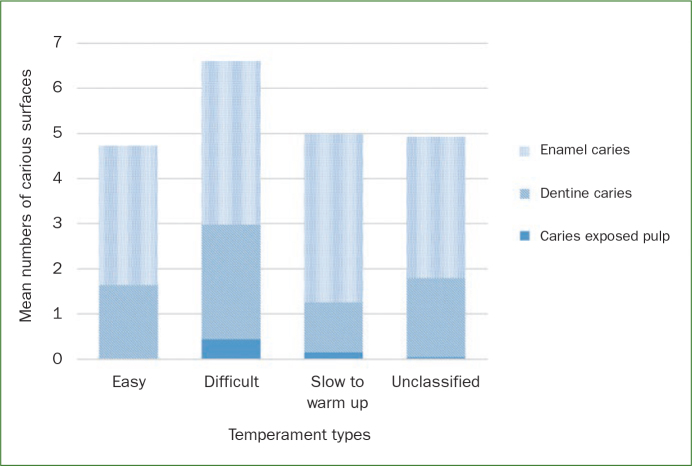
Total mean numbers of tooth surfaces including all three caries categories by child temperament type

Children with a difficult temperament type had the highest number of carious surfaces with the highest proportion of surfaces having d3 caries (caries-exposed pulp) (Fig 1). Children with an easy temperament type had about the same mean number of carious surfaces as the children in the slow-to-warm-up and mixed temperament groups; however, they had the lowest number of d3 caries. The differences were not statistically significant.

The score level categories were collapsed into two categories based on the distribution data in Table 2. Odds ratios for caries were calculated separately for each temperament trait (Table 3). The odds ratios were 1.80 and 1.74 for children with a high-score level of rhythmicity trait (arrhythmic) and approach/withdrawal trait (first response is withdrawal), compared to normal-score or low-score levels. Furthermore, children with normal-score or high-score levels of mood trait had 1.5 times the odds of having caries compared to those with a low-score level (positive/happy).

**Table 3:** Odds ratio and 95% confidence interval of having dental caries by temperament traits from univariable logistic regression analyses

Variable	Categories	Odds ratio	95% CI
Activity	0 = Low/Normal (Ref.) 1 = High	1.35	0.88, 2.06
Rhythmicity*	0 = Low/Normal (Ref.) 1 = High	1.80	1.15, 2.83
Approach/withdrawal*	0 = Low/Normal (Ref.) 1 = High	1.74	1.18, 2.57
Adaptability	0 = Low (Ref.) 1 = Normal/High	1.35	0.90, 2.03
Intensity	0 = Normal (Ref.) 1 = Low/High	0.73	0.43, 1.23
Threshold of responsive­- ness	0 = Low/normal (Ref.) 1 = High	1.28	0.86, 1.91
Mood*	0 = Low (Ref.) 1 = Normal/High	1.50	1.02, 2.21
Distractibility	0 = Low (Ref.) 1 = Normal/High	1.23	0.84, 1.80
Persistence or attention span	0 = Low/normal (Ref.) 1 = High	1.35	0.91, 2.01

Note: Ref.= reference group and * for p < 0.05.

The association between the mean milk-feeding frequency at night-time and the score levels of each temperament trait is presented in Table 4. Only an adaptability trait was found to be associated with feeding frequency at night. Children with normal-score or high-score levels of adaptability were fed significantly more frequently at night (p < 0.05) than those with a low-score level (very adaptable). Table 4 also shows that activity trait and approach/withdrawal trait were statistically significantly associated with regularity of oral cleaning habits by the children’s caregivers. Significantly more children with high-score levels for activity trait (very active) had regular oral cleaning than those with normal-score or low-score levels. However, significantly less children with a high-score level for approach/withdrawal trait (first response is withdrawal) had their oral cavity cleaned regularly.

**Table 4:** Average milk-feeding frequency and oral cleaning regularity by level of each temperament characteristic

Temperament characteristic	Level	Mean milk-feeding frequency at night*		Oral cleaning habits**		
		Times/night	p value	Regularly	Sometimes/ Never	p value
Activity	Low/Normal	2.90	0.70	38.2%	61.8%	0.04
	High	2.96		48.6%	52.4%	
Rhythmicity	Low/Normal	2.93	0.82	40.5%	59.5%	0.34
	High	2.89		43.2%	56.8%	
Approach/withdrawal	Low/Normal	2.91	0.94	45.6%	54.4%	0.03
	High	2.92		35.4%	64.6%	
Adaptability	Low	2.70	0.04	46.0%	54.0%	0.18
	Normal/High	3.01		39.3%	60.7%	
Intensity	Normal	2.84	0.61	36.1%	63.9%	0.31
	Low/High	2.93		42.3%	57.7%	
Threshold of responsiveness	Low/Normal	2.92	0.92	41.5%	58.5%	0.86
	High	2.91		40.7%	59.3%	
Mood	Low	2.92	0.98	43.0%	57.0%	0.55
	Normal/High	2.92		40.2%	59.8%	
Distractibility	Low	2.79	0.11	39.2%	60.8%	0.46
	Normal/High	3.00		42.6%	57.4%	
Persistence or attention span	Low/Normal	2.87	0.29	42.0%	58.0%	0.65
	High	3.01		39.9%	60.1%	

* t test; ** chi-squared test.

The results of the multivariable logistic regression model of dental caries status are presented in Table 5. Children with a high-score level of rhythmicity (arrhythmic), children with a high score of withdrawal (first response is withdrawal) and children with a normal score/high score of mood (normal to negative/unhappy) had a significantly higher chance of having caries (any level) after adjusting for regularity of oral cleaning habits and frequency of night feeding.

**Table 5:** Multivariable logistic regression model of having caries (including d1, d2 and d3)

Variable	Ref.	Adjusted OR	95% CI	p value
Oral cleaning habits	Regular	1.03	(0.69, 1.53)	0.89
Frequency of night feeding (time/night)	–	0.98	(0.86, 1.13)	0.82
Rhythmicity	High score	1.78	(1.12, 2.81)	0.02
Withdrawal	High score	1.76	(1.18, 2.63)	0.01
Mood	Normal score/High score	1.48	(0.996, 2.20)	0.052

## DISCUSSION

The present study is a longitudinal cohort study, thus the children were assessed for both temperament (at 12 months of age) and dental caries (at 18 months of age) at the same exact age for all subjects. Therefore, unlike other studies, this study did not have the problem regarding the effect of age variation such as older children having a higher chance to have more caries.

Children with a high score for rhythmicity trait indicated arrhythmic and irregular biological behaviours such as sleeping and feeding. These children had a significantly higher chance of having caries and had about two carious surfaces more than those with a normal/low score. These findings support a study by Santinath et al that reported statistically significant differences in the average number of nights the child slept through the night and the frequency of night waking episodes between groups of children with and without nursing caries.^16^ The question that came up first was whether these children were fed more frequently during the night waking. However, the present study did not find a statistically significant association between night feeding frequency and level of rhythmicity.

This study found that the children who withdrew when faced with a new person/stimulus (high-score level of approach-withdrawal trait) had a significantly higher chance of having dental caries. The withdrawal children are unresponsive to new stimuli and uncommunicative, possibly due to a lack of confidence or an inhibited character. The withdrawal children and shy children share some similar characteristics. In two published studies, shyness was associated with caries risk.^2,15^ Aminabadi et al reported that shyness was a predictor of prolonged breastfeeding.^2^ In the present study, the children with a high score for the trait of withdrawal were found to have significantly less regular oral cleaning habits, which possibly affected the dental caries status of these children.

The data revealed that the children with a normal or high score for a certain mood trait (unfriendly/unhappy/negative behaviour) tended to have a higher chance of having dental caries (p = 0.052). The issue of using feeding to regulate emotions is a concern for these children. One study reported that there was more consumption of sweet palatable foods in children whose mother frequently used food to regulate emotions.^4^ The results of our study where children with a normal/high score for mood having a higher chance of having caries and having more tooth surfaces with caries is consistent with the previous study.^2^ That study reported a higher sadness score in children with ECC compared to those without ECC.

There was also a relationship between the adaptability characteristic and night-feeding frequency. The adaptability characteristic demonstrates how easily a child can adjust to altered situations in a desired direction. A higher score level of adaptability, resisting change or guidance to behave in a desired way, was significantly related to high feeding frequency. One possible reason for this is that the child may use feeding as a tool to cope with the altered condition and their mother may also use feeding as a tool to control a child’s undesirable response to an altered condition. The study of Lee found that the most common response of mothers towards their baby’s crying was feeding.^10^ It seems, therefore, by implication that the baby who manifests a high intensity or high frequency of crying would be fed more frequently. Moreover, this study found that the children with a normal score or high score of adaptability had a higher probability of having caries. However, this relationship was not statistically significant.

Culture and child-rearing practices are possibly factors affecting the associations between temperament and dental caries among different races/ethnic populations. Co-sleeping is very common in Southeast Asia, including Thailand. In Thailand, only 0.1% of 3-month-old infants slept alone in a different room and 68.3% shared a bed with their parents. Compared to those parents who let their children fall asleep by themselves, co-sleeping parents would be more likely to over-react to any negative behaviours by their infants.^3^ Therefore, children who are not adaptable and do not sleep well when the place and time is changed would receive more intervention from their parents to help them sleep. Parents frequently use feeding to get their children to sleep during the night.

There were limitations of the study. Dental examination was conducted under natural light; this could lead to underestimation of children’s dental status. Also, temperament is an unmodifiable/very difficult to modify variable. Similar to other studies assessing temperament and outcome problem/disease, the results can only be used as indicator/risk assessment related factor. Therefore, findings of the present study can be used to identify both high risk individuals and the possible risk factors related to their temperament.

 A UK study reported grandparents used significantly more maladaptive feeding practices than parents; for example, using food to regulate the children’s emotions.^5^ Thus, children in an extended family with an unhappy mood would tend to be treated with more maladaptive feeding practices.

Rhythmicity and approach/withdrawal traits were significantly associated with a higher chance of having caries, after adjusting for oral cleaning habits and night feeding frequencies, in a multivariate analysis. It can be interpreted that there are other effects regarding the approach/withdrawal temperament trait other than oral cleaning habits and night feeding frequency. Further studies assessing effects of temperament on other factors relating to caries are needed.

## CONCLUSION

It can be concluded from the results of this study that arrhythmic children and children whose first response is withdrawal have higher chances of having caries. Additionally, positive/happy children tend to have lower chances of having caries. This finding helps clinicians understand about the importance of children’s caregivers. The three traits can also be used as caries determination factors.
